# A Multifunctional Interlocked Binder with Synergistic In Situ Covalent and Hydrogen Bonding for High‐Performance Si Anode in Li‐ion Batteries

**DOI:** 10.1002/advs.202302144

**Published:** 2023-08-16

**Authors:** Jae Hyuk Hwang, Eunji Kim, Eun Young Lim, Woohwa Lee, Ji‐Oh Kim, Inhye Choi, Yong Seok Kim, Dong‐Gyun Kim, Jin Hong Lee, Jong‐Chan Lee

**Affiliations:** ^1^ Advanced Materials Division Korea Research Institute of Chemical Technology 141 Gajeong‐ro, Yuseong‐gu Daejeon 34114 Republic of Korea; ^2^ School of Chemical and Biological Engineering and Institute of Chemical Processes Seoul National University 599 Gwanak‐ro, Gwanak‐gu Seoul 08826 Republic of Korea; ^3^ School of Chemical Engineering Pusan National University 2, Busandaehak‐ro 63beon‐gil, Geumjeong‐gu Busan 46421 Republic of Korea; ^4^ Advanced Materials and Chemical Engineering, KRICT School University of Science and Technology 217 Gajeong‐ro, Yuseong‐gu Daejeon 34114 Republic of Korea

**Keywords:** in situ cross‐linking, Li‐ion batteries, multifunctional binder, silicon anodes, thiourea polymer network

## Abstract

Silicon has garnered significant attention as a promising anode material for high‐energy density Li‐ion batteries. However, Si can be easily pulverized during cycling, which results in the loss of electrical contact and ultimately shortens battery lifetime. Therefore, the Si anode binder is developed to dissipate the enormous mechanical stress of the Si anode with enhanced mechanical properties. However, the interfacial stability between the Si anode binder and Cu current collector should also be improved. Here, a multifunctional thiourea polymer network (TUPN) is proposed as the Si anode binder. The TUPN binder provides the structural integrity of the Si anode with excellent tensile strength and resilience due to the epoxy‐amine and silanol‐epoxy covalent cross‐linking, while exhibiting high extensibility from the random coil chains with the hydrogen bonds of thiourea, oligoether, and isocyanurate moieties. Furthermore, the robust TUPN binder enhances the interfacial stability between the Si anode and current collector by forming a physical interaction. Finally, the facilitated Li‐ion transport and improved electrolyte wettability are realized due to the polar oligoether, thiourea, and isocyanurate moieties, respectively. The concept of this work is to highlight providing directions for the design of polymer binders for next‐generation batteries.

## Graphical abstract

A multifunctional interlocked thiourea polymer network (TUPN) binder with in situ covalent and hydrogen bonding for Si anode in Li‐ion batteries is presented. Based on enhanced mechanical properties through covalent cross‐linking and hydrogen bonding, thiourea‐based physical interlocking, and facilitated Li‐ion transportation via polar moieties, stable and high‐performance Si anode in Li‐ion batteries is realized.

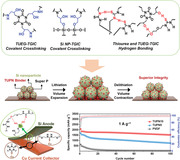

## 1 Introduction

Recently, Li‐ion batteries (LIBs) have rapidly developed as promising technologies in energy storage devices, such as electric vehicles and smart grids, while reducing the dependence on fossil fuels.^[^ ^1^, ^2^, ^3^ ^]^ However, with the increasing use of electronic devices that require higher energy densities, conventional graphite anodes (372 mAh g^−1^) based on intercalation chemistry are faced with significant limitations.^[^ ^4^, ^5^ ^]^ Accordingly, with a theoretical capacity (3579 mAh g^−1^ at lithiation to Li_15_Si_4_) approximately ten times higher than graphite, Si has garnered significant attention as a high‐capacity electrode material for next‐generation LIBs. Moreover, Si has stimulated several studies owing to its low operating voltage, non‐toxicity, abundant natural sources, and low cost.^[^ ^6^ ^]^ Despite the high potential of Si materials, the large volume change (≈300%) of Si particles during the lithiation/delithiation process has hampered their practical applications.^[^ ^7^ ^]^ The continuous volume change triggers the pulverization of Si particles and delamination of the electrode material from the current collector.^[^ ^8^ ^]^ These problems not only reduce electrical contact of the active materials but also form an unstable solid electrolyte interface (SEI) layer on the Si particle surface, thereby resulting in rapid capacity fading and poor cycle life of the Si anode. To address the aforementioned challenges, several strategies have been proposed for nanostructured Si materials, such as core‐shell,^[^ ^9^ ^]^ yoke‐shell,^[^ ^10^ ^]^ nanowire,^[^ ^11^ ^]^ nanotube,^[^ ^12^ ^]^ etc. The nanoscale design effectively withstands the internal stress caused by the volume expansion and provides a short transport path for Li‐ions, thereby improving the cycling stability. Nevertheless, nanoscale Si particles with a high surface area would promote the formation of SEI layers during the initial cycling, leading to an increase in the irreversible capacity. In addition, the problem of production cost for nanoscale Si remains because of its complex manufacturing process.

Another perspective and facile approach for high‐performance Si anodes is the development of novel polymer binders.^[^ ^13^, ^14^, ^15^ ^]^ A binder suitable for coupling active materials preserves the structural integrity of an electrode and plays an important role in stabilizing the electrolyte‐electrode interface.^[^ ^16^ ^]^ Linear poly(vinylidene fluoride) (PVDF) is a representative commercial binder that possesses weak van der Waals interactions. Hence, PVDF is insufficient in alleviating the rapid volume expansion of Si particles and dissipating internal stress.^[^ ^17^ ^]^ Poly(acrylic acid) (PAA) and carboxymethyl cellulose binders are commonly used as Si anode binders due to the many ─COOH groups and ─OH groups to form strong hydrogen bonds.^[^ ^14^ ^]^ However, these binders exhibit high tensile strength but very low elongation, so it is challenging to endure large volumetric changes due to continuous lithiation/delithiation processes.^[^ ^18^, ^19^ ^]^ Recently, polymeric binders with various functional groups have been explored.^[^ ^20^, ^21^ ^]^ The criteria required for an ideal polymer binder are as follows: 1) The binder should exhibit high mechanical strength and flexibility to accommodate the volume changes of Si particles. To handle enormous stresses, it is effective to improve the mechanical properties by designing the 3D structure of a cross‐linked network.^[^ ^22^ ^]^ 2) It is important to have high interfacial stability between the binder, active material, and Cu current collector. A polymer binder with polar functional groups (─OH, ─NH, and ─COOH) can form a hydrogen bond with a silanol group (Si─OH) on the Si surface, thereby improving the interfacial adhesive force.^[^ ^23^ ^]^ In particular, the introduction of a direct covalent bond between the functional group of the binder and Si particles makes it possible to prepare a stable Si anode by supplementing the insufficient strength of the secondary bond.^[^ ^24^ ^]^ 3) High ion and electron conductivities are required. Certain structures, including polyethylene oxide or polyethyleneimine, can accelerate the transfer of Li‐ions via a coordination bond of a lone pair electron, leading to remarkably improved rate performance and cycle reversibility.^[^ ^25^, ^26^ ^]^ In turn, high‐performance Si anodes depend on the design of a novel polymer binder with desirable properties.

**Scheme 1:**
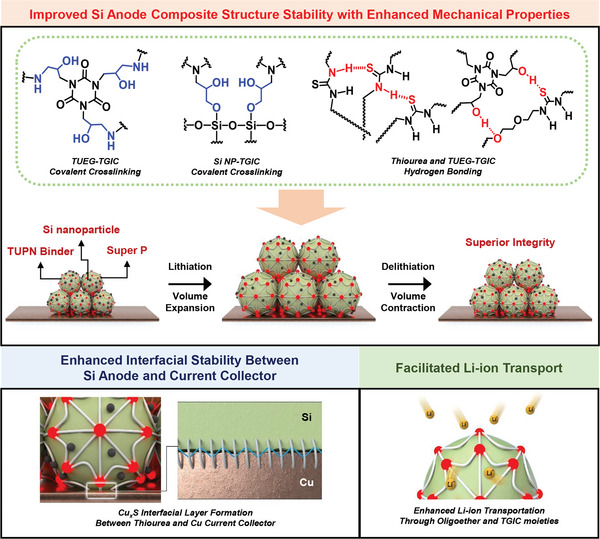
Schematic of chemical structures and illustrations of TUPN binder with enhanced electrochemical properties.

Recently, Aida et al. reported a seminal study on poly(ether‐thiourea)s with triethylene glycol (TUEG) as a spacer with self‐healing and robust properties.^[^ ^27^ ^]^ These thiourea‐based polymers have been introduced into electrochemistry and applied as self‐healing Si anode binders.^[^ ^28^, ^29^, ^30^, ^31^, ^32^ ^]^ However, the polymeric binders that are not three‐dimensionally cross‐linked have the potential to dissolve in the electrolyte owing to their low cross‐linking density. Therefore, research on multifunctional polymeric binders based on three‐dimensionally cross‐linked thiourea moieties is still required. Herein, we present a multifunctional thiourea polymer network (TUPN) binder, considering the challenges of pulverization of the Si particles and delamination of the electrode from the current collector (**Scheme** **1**). The thermally in situ cross‐linked TUPN binder has two features. First, the OH group of the Si nanoparticles and amine groups of TUEG were thermally in situ cross‐linked with epoxy groups of triglycidyl isocyanurate (TGIC). Three‐dimensionally cross‐linked by TGIC cross‐linker, the TUPN binder exhibits excellent mechanical properties based on high tensile strength and sufficient resilience, while exhibiting high extensibility via a random coil network of TUEG chains with hydrogen bonds. In addition, forming covalent bonds between silanol groups of Si nanoparticles and epoxy groups of TGIC leads to reinforced silicon‐binder bond strength. Hence, during the repeated charging and discharging processes, it is possible to withstand the volume expansion of Si and achieve the anti‐pulverization of Si nanoparticles due to the Si‐TGIC covalent bond formation. Second, the adhesion between the TUPN binder and Cu current collector was significantly increased by the thiourea and isocyanurate moieties. The strong interactions of thiourea–copper and urea–copper can prevent the delamination of the electrode from the current collector.^[^ ^29^, ^31^, ^32^ ^]^ In particular, TUPN contains a large amount of thiourea and triethylene glycol units through the TUEG oligomer chains, while also containing isocyanurate moieties from TGIC. Due to oligoether, thiourea, and isocyanurate groups, TUPN binder has high polarity. Therefore, the wetting of the electrolyte is enhanced, leading the increased Li‐ion concentration, and the Li‐ion conductivity also improves. Based on versatile characteristics, the Si anode with the TUPN binder exhibits a highly desirable rate capability (785.4 mAh g^−1^ at a high current rate of 5.0 A g^‒1^) and superior cycling stability (1530.1 mAh g^−1^ after 100 cycles).

## 2 Results and Discussion

**Figure 1:**
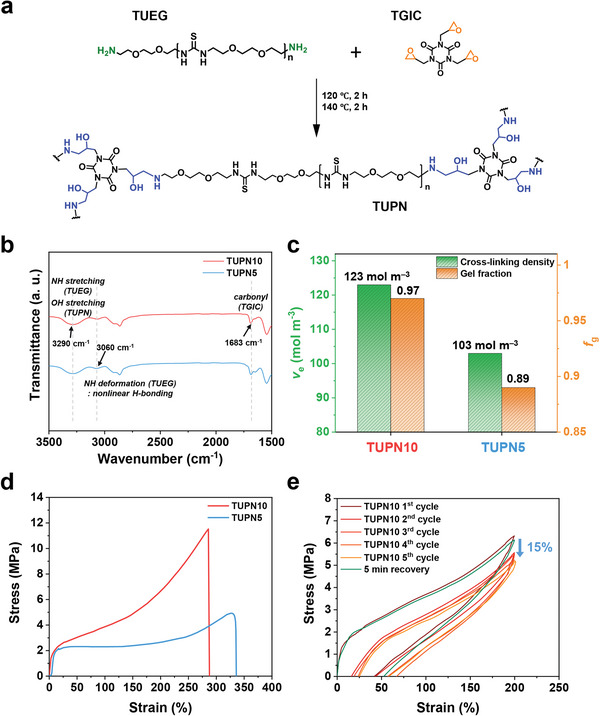
Design and characterization of three‐dimensionally cross‐linked TUPN. a) Chemical structures of TUEG and TGIC, and synthetic process of TUPNs. b) FT‐IR spectra of TUPN10 and TUPN5. c) Cross‐linking density and gel fraction of TUPN10 and TUPN5, respectively. d) Representative stress−strain curves of TUPN10 and TUPN5 at room temperature (25 ± 1 °C) and a strain rate of 0.008 s^−1^. e) Cyclic stress–strain curves of TUPN10 (from 1st to 5th cycle and after recovery for 5 min at 25 °C) at room temperature (25 ± 1 °C) and a strain rate of 0.008 s^−1^.

**Figure** **1**a illustrates the chemical structure of three‐dimensionally cross‐linked TUPN comprising NH_2_ group‐terminated TUEG oligomers and epoxy group‐terminated TGIC cross‐linkers. The TUEG oligomer (TUEG#, where # indicates the molecular weight of TUEG) used for TUPN polymerization was synthesized by the polycondensation of 1,2‐bis(2‐aminoethoxy)ethane (EGDA) and 1,1′‐thiocarbonyldiimidazole (TCDI) (Figure S1, Supporting Information). The molecular weight of TUEG can be controlled by the feed ratio of EGDA and TCDI. The synthesized TUEG2600 and TUEG4300 were verified via ^1^H nuclear magnetic resonance (NMR) (Figure S2, Supporting Information). For characterization, a TUPN film was prepared using the solution casting method. TUEG and TGIC in dimethylformamide (DMF) were cast onto a Teflon substrate and dried at 50 °C overnight. After drying at 80°C under vacuum conditions for 1 h, the resultant film on a Teflon plate was cured at 120 and 140 °C for 2 h, respectively, under the same vacuum conditions. The cured TUPNs were designated as TUPN*x*, where *x* denotes the weight percentage (wt.%) of TGIC. The molar feed ratio of the NH_2_ group in TUEG to the epoxy group in TGIC ([amine]:[epoxy]) was fixed at 1:1. Due to cross‐linking between the primary amine and epoxy groups, TUEG is consequently polymerized into TUPN. Since the cross‐linking density of TUPN depends on the chain length of TUEG, the cross‐linking density of TUPN10 polymerized from TUEG2600 is higher than that of TUPN5 polymerized from TUEG4300 (Figure S3 and Table S1, Supporting Information).

The covalent cross‐linking of the TUPNs was verified by Fourier transform infrared (FT‐IR) spectroscopy, gel fraction, and cross‐linking density. The OH and NH stretching peak (3290 cm^−1^) of the TUPN, NH deformation vibration peak (3060 cm^−1^) of the nonlinearly hydrogen‐bonded thiourea units, and carbonyl peak (1683 cm^−1^) from TGIC are presented in Figure 1b and Figure S4 (Supporting Information).^[^ ^27^, ^33^, ^34^ ^]^ Since the gel fraction (*f*_g_) represents the mass fraction of the polymer network resulting from a network‐forming polymerization or crosslinking process,^[^ ^35^ ^]^ we performed gel fraction tests on the TUPNs in both the electrolyte and DMF (Table S1, Supporting Information). As illustrated in Figure 1c, the resulting gel fraction of TUPN10 (0.97) is 11% higher than that of TUPN5 (0.89). Cross‐linking density (*ν*_e_) refers to the number of crosslinks per unit volume in a polymer network.^[^ ^35^ ^]^ To determine the cross‐linking density, dynamic mechanical analysis (DMA) was performed by analyzing the storage modulus (*E*') of the rubbery plateau region (Figure S5, Supporting Information). TUPN10, comprising shorter TUEG chains than TUPN5, formed a denser network structure, resulting in a 19% higher cross‐linking density (Table S1, Supporting Information). Thermal analyses were conducted to confirm the thermal stabilities of TUPNs. From thermogravimetric analysis (TGA), the thermal decomposition temperatures of TUPN10 and TUPN5 were 274.8 and 272.3 °C, respectively, showing excellent thermal stability (Figure S6, Supporting Information). In addition, as illustrated in Figure S7 (Supporting Information), the amorphous nature of TUPN was verified by the absence of a melting point peak over the temperature range via differential scanning calorimetry (DSC).

TUPN shows outstanding tensile strength, sufficient resilience, and high extensibility from TUEG‐TGIC covalent bonding, thiourea physical cross‐linking, and randomly coiled TUEG chains, respectively.^[^ ^33^ ^]^ We investigated the mechanical properties to evaluate whether TUPN can withstand high volume change (300‒400%) and the pulverization of Si that occurs between lithiation and delithiation. As shown in Figure 1d, TUPN10, comprising a relatively shorter TUEG chain, presented a smaller elongation (286%) than TUPN5. However, owing to its high cross‐linking density, TUPN10 exhibited an ultimate tensile strength (11.5 MPa) and a toughness (15.7 MJ m^−3^) four times and almost twice that of TUPN5, respectively (Table S2, Supporting Information).^[^ ^36^ ^]^ However, TUPN23, consisting of relatively shorter TUEG1000 chains, demonstrated improved tensile strength, albeit with a significant reduction in elongation attributable to the presence of short random coil chains. (Figure S8, Supporting Information). To approach the practical applications, we also conducted uniaxial tensile tests on TUPNs after electrolyte swelling for 24 h (Figure S9, Supporting Information). The breaking strength values of TUPN10 and TUPN5 decreased to ≈1.92 and 0.83 MPa after swelling in the electrolyte. Additionally, the elongation at break values decreased to 83 and 112%, respectively. The decrease in mechanical properties can be attributed to the significant swelling of the TUPNs caused by the electrolyte.^[^ ^37^, ^38^ ^]^ Due to the presence of oligoether, thiourea, and isocyanurate moieties, the TUPN can sufficiently uptake electrolytes, thus leading to the degradation of its mechanical properties. However, electrolytes are not injected excessively during the cell assembly of batteries in general, as in the case of the swelling test in this manuscript. Therefore, TUPN‐based Si anodes may not show excessively lowered mechanical properties in assembled cells. In addition, the Si anode is a composite material comprising Si nanoparticles, TUPN, and Super P, with the TUPN accounting for 20 wt.% of the electrode's content. Since the Si anode has a low content of TUPN, the electrode composite may not swell excessively in the electrolyte. Moreover, as the TGIC cross‐links with some Si nanoparticles, the mechanical properties of the composite electrode in the electrolyte are assumed to be improved, unlike the swelling conditions with TUPN film only. TUPN, composed of thiourea, oligoether, and isocyanurate moieties, exhibits high polarity (Figure S10, Supporting Information). Because isocyanurate moieties in TUPN have polar polymer‐solvent interactions with ethylene carbonate (EC) and diethyl carbonate (DEC) electrolytes,^[^ ^39^ ^]^ TUPN10 (62.4%) contains more electrolytes than TUPN5 (50.0%) with enhanced electrolyte wettability (Figure S11, Supporting Information). While linear TUEG is soluble in the electrolyte, cross‐linked TUPN exhibits high gel fraction values. Therefore, the swelling ratio of TUPN over time tended to be highly saturated.

Because the volume of Si nanoparticles shrinks again during the delithiation process, a TUPN cyclic tensile test was performed to verify the resilience of the binder material. After the first loading‐unloading cycle within a strain limit of 200%, TUPN10 exhibited a residual, temporary strain of ≈50%, which increased slightly up to ≈63% after five consecutive cycles (Figure 1e). Even after five cycles, the stress value decreased by 15% with maintained sufficient resilience. After allowing 5 min of rest at room temperature (23 °C), the pre‐stretched TUPN10 fully recovered its original shape and mechanical properties. Therefore, we considered that TUPN10, which is more highly cross‐linked than TUPN5, may provide excellent resilience (Figure S12, Supporting Information).

**Figure 2:**
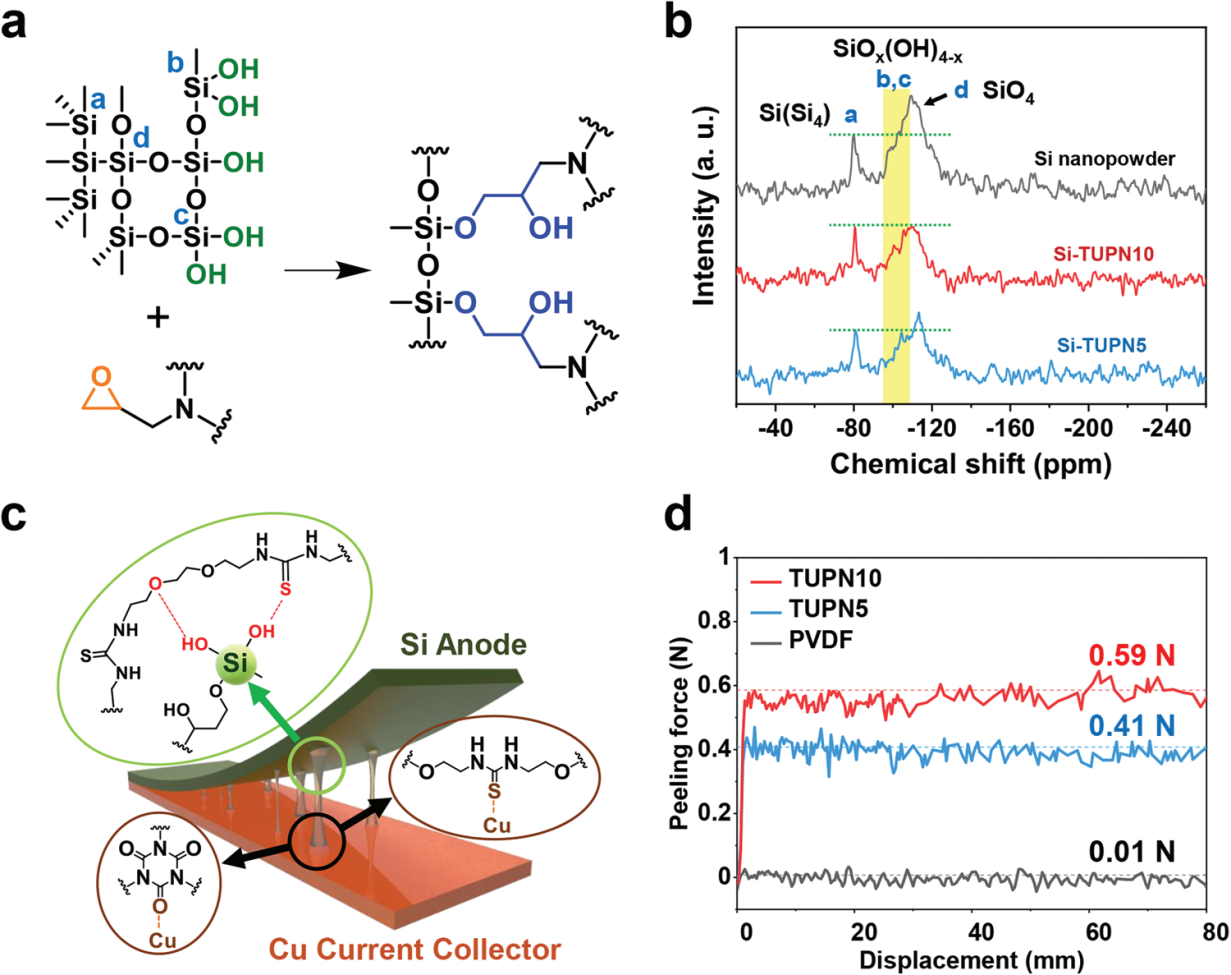
Characterization of Si‒TUPN covalent bonds and TUPN‒Cu interaction. a) Mechanism of the reaction between ‒OH groups of Si nanoparticle and epoxy groups of TGIC. b) Solid‐state ^29^Si MAS NMR spectra of Si nanopowder, Si‒TUPN10, and Si‒TUPN5. c) Schematic illustrations of adhesive TUPN binder between Si anode and Cu current collector. d) Peeling force plot and average peeling force comparison of PVDF, Si‒TUPN10, and Si‒TUPN5 from the peeling test.

In addition to the excellent mechanical and thermal properties of TUPN, thermal in situ polymerization of TUPN was performed to conduct an electrochemical analysis. We prepared a homogeneous slurry by mixing the Si powder, TUEG, TGIC, and conductive carbon, and then coated it on the copper current collector. The drying process was simultaneously performed in two steps (120 and 140°C for 2 h, respectively) with in situ polymerization. TGIC and Si nanoparticles can react to form covalent bonds during thermal in situ polymerization. Cho et al. first reported the formation of a covalent bond between a binder and a Si nanopowder using a condensation reaction between a silanol group and carboxyl group in PAA.^[^ ^24^ ^]^ Considering that the Si‒PAA covalent bond restrains the high volume change in Si nanoparticles and prevents the destruction of the electrical network during cycling, we adopted the reaction of the silanol groups on the silica surface with the epoxy groups.^[^ ^40^, ^41^ ^]^ As shown in **Figure** **2**a, the silanol group on the Si nanoparticle surface reacts with the epoxy group of the TGIC cross‐linker to form a Si─O─C bond during the thermal in situ polymerization. During the thermal polymerization, Si anode (Si─TUPN) with covalent bond linkages between Si‒TGIC‒TUEG forms from the doctor‐bladed mixtures. We verified the Si‒TGIC covalent bond via the ^29^Si magic‐angle spinning (MAS) solid NMR analysis of Si‒TUPN. Figure 2b presents the ^29^Si MAS NMR spectra of the silicon composite electrode and Si nanopowder. The peak intensity of OH group on the surface of the Si nanopowder, approximately from −95 to −107 ppm,^[^ ^42^ ^]^ decreased in Si‒TUPN10 and Si‒TUPN5 after the thermal in situ polymerization. The ─OH peak in Si‒TUPN10 is further reduced because TUPN10 has more epoxy groups that can react with Si nanopowder than Si‒TUPN5. Hence, the covalent bond formation on Si‒TUPN10 may induce an enhanced bonding strength between Si and the binder.

To further investigate the Si─O─C bond formation of the Si‐TUPNs, we fabricated Si‐TUPN samples (Si and binder materials in a weight ratio of 75:25 without Super P) and confirmed the Si─O─C bond through FT‐IR analysis. As shown in Figure S13 (Supporting Information), Si‐TUPN10 and Si‐TUPN5 exhibited Si─O─C bond shoulder peak at 1133 cm^‒1^.^[^ ^43^ ^]^ To confirm whether the reaction between silanol and epoxy affects the cross‐linking network of TUPN, we fabricated Si‐TUPN composite (Si and binder materials in a weight ratio of 75:25 without Super P) through thermal in situ polymerization and conducted a gel fraction test. As shown in Table S3 (Supporting Information), Si‐TUPN10 and Si‐TUPN5 showed very high gel fraction values. Since the high reactivity of the amine and epoxy group than the silanol and epoxy group,^[^ ^44^ ^]^ we assumed that TUPNs could retain their cross‐linked network structures while establishing suitable covalent bonding with Si particles.

The TUPN binder can also improve the interfacial stability of the current collector. Due to the rubber–metal binding mechanism, thiourea moieties of the TUPN binder can be interlocked to the Cu current collector as Cu_x_S with a dendrite form.^[^ ^45^, ^46^ ^]^ Cu current collector and sulfur can form the Cu_x_S interaction by reacting with Cu during in situ polymerization and can increase the binding force with the Cu current collector.^[^ ^29^, ^31^, ^32^ ^]^ In addition, because the isocyanurate moiety comprising triurea moieties favors the adsorption to Cu and CuO, it can increase the adhesion force via physical bonding.^[^ ^47^ ^]^ Therefore, thiourea‐based TUPN binders are expected to have better adhesion to Cu current collectors than previously reported polyurea and polyurethane‐based binders, which had excellent mechanical properties.^[^ ^48^, ^49^ ^]^ Figure 2c presents the schematic illustrations of the adhesive TUPN binder between the Si anode and Cu current collector. The 180° peeling test was conducted to measure the adhesion strength of the Si electrode and Cu current collector interface.^[^ ^50^ ^]^ Figure 2d presents the adhesion results, showing the increased adhesion force between the in situ polymerized Si‒TUPN and Cu current collector. Si‒TUPN10 and Si‒TUPN5 exhibit greater peeling force (0.59 and 0.41 N, respectively) than commercial PVDF (0.01 N). Among the Si‒TUPNs, Si‒TUPN10 has 6.7 mol.% fewer thiourea moieties than Si‒TUPN5; however, it exhibits a higher peeling force because Si‒TUPN10 has 59 mol.% more urea moieties than Si‒TUPN5. We assumed that the strongly adhesive Si‒TUPN10 may prevent delamination from the Cu current collector while realizing interfacial and cycling stability.

**Figure 3:**
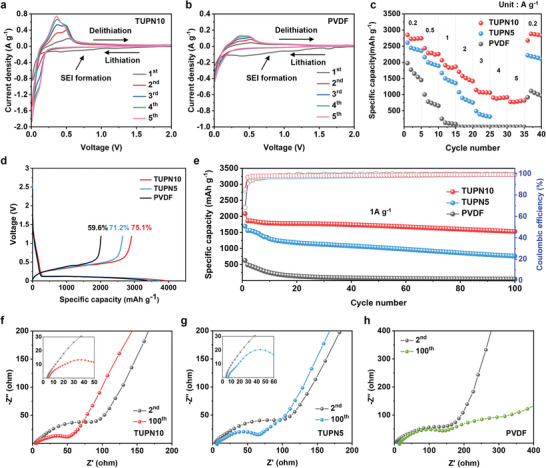
Cyclic voltammetry of the Si anode with a) TUPN10 and b) PVDF binders. c) Rate capability at various rates from 0.2 to 5.0 A g^‒1^. d) Initial charge/discharge cycling and coulombic efficiency of the Si anodes with PVDF, TUPN5, and TUPN10 binders, under a current density of 0.1 A g^‒1^, respectively. e) cycling performance at 1.0 A g^‒1^ of the Si anode with PVDF, TUPN5, and TUPN10 binders. Nyquist plots of the Si anode with a) TUPN10, b) TUPN5, and c) PVDF binders after 2nd and 100th cycles.

The effect of TUPN binders on the electrochemical performance of the Si anode was evaluated by cyclic voltammetry (CV) for the initial five cycles, with a scan rate of 0.1 mV s^‒1^. As illustrated in **Figure** **3**a, during the first cycling, a wide peak was observed at approximately 0.5‒1.2 V, which could be attributed to the formation of SEI layers resulting from the decomposition of electrolytes.^[^ ^51^ ^]^ In subsequent cycles, the CV profile of the Si anode exhibited a reduction peak at 0.11 V and two oxidation peaks at ≈0.36 and 0.50 V, respectively. The former peak corresponded to a Li‒Si alloying process, while the latter pronounced peaks were attributed to a dealloying reaction.^[^ ^51^, ^52^ ^]^ The gradual increase in the peak intensity over the five scan steps was related to the activation process of the Si anode.^[^ ^52^ ^]^ Clearly, the cell with the TUPN10 binder exhibited a sharper and significantly more distinct current peak than those with the TUPN5 and PVDF binders (Figure S14, Supporting Information; Figure 3b), indicating that the TUPN10 binder was able to improve the utilization of Si active materials and activity of the electrode.

As illustrated in Figure 3c, the rate capability of the Si anode with various binders was investigated at current densities of 0.2–5.0 A g^‒1^. The Si anode with the TUPN5 binder delivered a relatively high specific capacity of 2605.2 mAh g^‒1^ at a current density of 0.2 A g^‒1^, but was unable to maintain this high value at higher current densities over 4.0 A g^‒1^. Similarly, the Si anode with the PVDF binder suffered from significant specific capacity fading. In contrast, the Si anode with the TUPN10 binder not only exhibited a high initial specific capacity of 2849.3 mAh g^‒1^, but also achieved the highest specific capacities at all current densities applied, retaining a stable specific capacity of 785.4 mAh g^‒1^ even under the harsh condition of 5.0 A g^‒1^. Notably, TUPN23 exhibited poor electrochemical performance, possibly owing to its excessively dense network structure (Figure S15, Supporting Information). Therefore, we presume that appropriately cross‐linked TUPN10 has excellent mechanical properties and forms sufficient covalent bonds with Si to prevent the pulverization of Si. Figure 3d shows the Initial charge/discharge cycling and coulombic efficiency (CE) of the Si anodes with PVDF, TUPN5, and TUPN10 binders at a current density of 0.1 A g^‒1^, respectively. The initial coulombic efficiencies (ICE) of the Si anodes with PVDF, TUPN5, and TUPN10 binders under a current density of 0.1 A g^‒1^ were found to be 59.6, 71.2, and 75.1%, respectively, revealing the high reversibility of the Si anode with the TUPN10 binder. We suggested that this result is due to the strong adhesion and mechanical properties of TUPN binders.^[^ ^53^ ^]^ TUPN forms a cross‐linked structure with Si particles and increases adhesion through interaction with the Cu current collector. In addition, TUPN with excellent mechanical properties can accommodate huge volume changes of Si anodes. Since TUPN10 exhibits more outstanding mechanical properties and is more polar than TUPN5, TUPN10 can induce reversible Li‐ion insertion and extraction at the Si anode during the volume changes of the Si anode, resulting in high ICE.^[^ ^53^ ^]^ Additionally, we fabricated electrodes with varying thicknesses and confirmed the areal capacity based on the mass loading of Si nanoparticle.^[^ ^6^, ^54^, ^55^, ^56^ ^]^ As the thickness of the electrode increases in tandem with the Si loading, the areal capacity experiences proportional enhancement, thereby signifying the effective binding capabilities of TUPN (Figure S16, Supporting Information). We considered that the robust adhesion and mechanical properties of the TUPN10 binder facilitated a consistent enhancement in the areal capacity and ensured stable cycling performance without significant degradation in a specific capacity. Consequently, relying on the TUPN binder, we anticipate a proportional increase in the areal capacity, even when an increased quantity of Si is introduced to the electrode.

To further evaluate the cycling stability and CE of the Si anode with various binders, the cells were cycled for 100 cycles. As shown in Figure 3e, the Si anode with the PVDF binder experienced rapid capacity decay at a current density of 1.0 A g^‒1^, and reached a specific capacity of merely 137.1 mAh g^‒1^ after 20 cycles. While the Si anode with the TUPN5 binder achieved a relatively higher initial capacity of 1695.1 mAh g^‒1^ than that of the PVDF binder, its cycling stability (44.9% of retention) was still unsatisfactory during 100 cycles. As expected, the Si anode with the TUPN10 binder exhibited outstanding cycling stability, delivering a high initial specific capacity of 2087.7 mAh g^‒1^ and retaining the highest specific capacity of 1530.1 mAh g^‒1^ (73.3% of retention) with a CE of 99.1%, even after 100 cycles. Even under charge‐discharge rates twice as fast as Figure 3e, the Si anode with TUPN10 binder still exhibited consistent and reasonable specific capacities over 200 cycles, without any significant capacity fading (Figure S17, Supporting Information). To confirm practical applicability of the TUPN10 binder, we prepared a full‐cell configuration utilizing Ni_0.6_Co_0.2_Mn_0.2_ (NCM_622_) as the cathode and conducted a galvanostatic cycling test to evaluate the electrochemical performance of the Si anode employing the TUPN10 binder. As shown in Figure S18 (Supporting Information), the full‐cell incorporating the Si anode with the TUPN10 exhibited reasonable specific capacity values, indicating the significant role of the TUPN10 binder irrespective of the cell configuration. Even though the cycling stability of the full‐cell was observed to be somewhat lower than that of the half‐cell configuration, we believe that optimizing the fabrication process has the potential to further enhance the long‐term cyclability of the Si‐TUPN anode‐based full‐cell. It should be noted that the cyclic stability of the full‐cell can be influenced by the various fabrication parameters, including electrode pressing, additives of electrolyte, and precise control of N/P ratio.^[^ ^57^, ^58^ ^]^ Therefore, we speculate that the improved electrochemical performance of TUPN is due to the robust adhesion and mechanical properties of the TUPN10 binder, providing further evidence of the structural integrity achievement. In particular, TUPN10, which is appropriately cross‐linked, has excellent tensile strength, extensibility, and resilience. Therefore, TUPN10 prevents Si pulverization, effectively preserves the structural integrity of the Si anode, and facilitates the transport of ions and electrons, thereby leading to excellent electrochemical performance.

In order to investigate the electrochemical kinetics and interfacial properties of Si anodes with various binders, electrochemical impedance spectroscopy (EIS) was conducted after 2 and 100 cycles. As presented in Figure 3f‒h, the Nyquist plots were composed of two semicircles in the high‐ and middle‐frequency regions related to the resistance of the SEI layer (*R*_SEI_) and charge transfer (*R*_CT_), as well as inclined lines in the low‐frequency region corresponding to the diffusion of Li‐ions (*W*_s_).^[^ ^22^ ^]^ The equivalent circuits corresponding to this model are provided in Figure S19 (Supporting Information). After two cycles of the activation process, the Si anode with TUPN10 binder showed relatively low *R*_SEI_ (5.9 Ω) and *R*_CT_ (54.3 Ω) values, indicating effective volume change accommodation and mitigation of parasitic electrolyte decomposition reactions owing to the mechanically robust TUPN10 network structure. Furthermore, the Si anodes with the TUPN10 binder exhibited a slight reduction in semicircles after 100 cycles. We speculated that the high Si‐binder adhesive force and stable electrode‐electrolyte interface of the TUPN binders were able to facilitate the maintenance of electrical contact between the Si active material and conductive carbon, leading to superior electrochemical performance. In contrast, the Si anode with the PVDF binder displayed a significant increase in the two semicircles upon cycling, which could be associated with the insufficient mechanical properties of the PVDF binder, causing the formation of a thick SEI layer and breaking the electrical contact due to the continuous pulverization of Si particles.

As the charging and discharging proceed in the wet state of the electrolyte in the battery binder, TUPN, which does not dissolve in the electrolyte with a maintained network structure, could improve Li‐ion conductivity via sufficient electrolyte uptake (Figure S11, Supporting Information). We performed calculations to determine the diffusion coefficient of Li‐ions (*D*_Li_^+^) based on the EIS low‐frequency region of the Si anode with different binders using Equations (1) and (2):^[^ ^59^, ^60^ ^]^

(1) $$D_{\mathrm{Li}}^{+}=R^{2}T^{2}/2A^{2}n^{4}F^{4}C^{2}\sigma^{2}$$

(2) $$Z^{\prime }=R_{0}+R_{\mathrm{CT}}+\sigma \omega^{-1/2}$$

where *R* is the gas constant, *T* is the absolute temperature, *A* is the electrode surface area, *n* is the number of electrons per molecule during the redox reaction, *F* is the Faraday constant, *C* is the Li‐ion concentration in the electrolyte, *σ* is the Warburg coefficient, *Z*’ is the real part resistance, *R*_0_ is the bulk resistance of the electrolyte, and *ω* is the angular frequency. Figure S20 (Supporting Information) shows plots of the linear relationship between *Z*’ and *ω* ^‒1/2^, in which σ is determined from the slope of the fitted line. The calculated *D*_Li_^+^ values are listed in Table S4 (Supporting Information). As expected, the Si anode with the TUPN10 binder exhibited the highest *D*_Li_^+^ value of 2.38 × 10^‒21^ cm^2^ s^‒1^, demonstrating the facilitated redox reaction kinetics and Li‐ion transport in the Si anode.

To better understand the enhanced Li‐ion transport properties of the TUPN10 binder, we examined the ionic conductivity and wettability of the polymer films. The ionic conductivity was measured by EIS using coin cells assembled with different binder films inserted between two stainless steel spacers.^[^ ^6^, ^61^ ^]^ When assembling the coin cell, an excessive amount of electrolyte was added and the film was wetted for 24 h, and then the ion conductivity was measured. As illustrated in Figure S21 (Supporting Information), the ionic conductivities of the TUPN10 and TUPN5 films were 9.75 × 10^‒6^ and 1.81 × 10^‒6^ s cm^‒1^, respectively, which are approximately 17.4 and 3.23 times higher than that of the PVDF film (5.60 × 10^‒7^ s cm^‒1^). The discrepancy in Li‐ion transport observed between the TUPN and PVDF films could be attributed to the presence of polar moieties in TUPN, including oligoether, thiourea, and isocyanurate groups. We also investigated the contact angle of the pristine TUPN10 and TUPN5 films (Figure S10, Supporting Information). The TUPN10 film exhibited a smaller contact angle value of 66.7° than that of the TUPN5 film (86.2°). TUPN10 has more polar functional groups than TUPN5. The more polar TUPN10 has better electrolyte uptake and better swelling, as discussed in Figure S11 (Supporting Information). Although TUPN23 is highly polar, swelling via electrolyte uptake is challenging due to the dense cross‐linking state. As shown in Figure S22 (Supporting Information), TUPN23, which does not sufficiently uptake the electrolyte, shows low ionic conductivity. Hence, the TUPN10 binder may facilitate more Li‐ion transport than TUPN5 owing to its high compatibility with the electrolyte.

**Figure 4:**
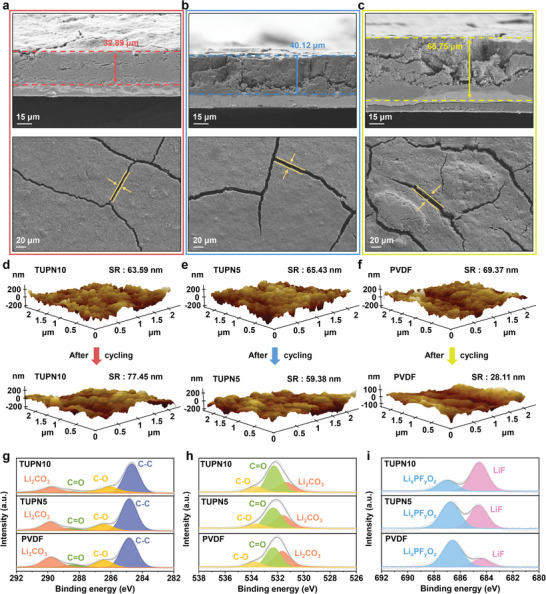
Cross‐section and top surface SEM image of the Si anodes with a) TUPN10, b) TUPN5, and c) PVDF binders after 100 cycles. AFM images of the Si anode surface with d) TUPN10, e) TUPN5, and f) PVDF binders before and after cycling. g) C 1s, h) O 1s, and i) F 1s XPS spectra of the Si anodes with various binders after 100 cycles.

In order to determine the effect of the TUPN binder on suppressing the expansion of the Si anode, the surface images and thickness changes of Si anodes with various binders after cycling were investigated. The initial thickness of the electrode was approximately 15 µm, which is consistent for all three Si anodes (Figure S23, Supporting Information). After 100 cycles, the Si anode with the TUPN10 binder expanded to a thickness of 32.89 µm (121.8% of the initial thickness) with only minor cracks present on the surface of the electrode (**Figure** **4**a). The thickness of the electrode using the TUPN5 binder was observed to be 40.12 µm, slightly higher than that of the TUPN10 binder (Figure 4b). We considered that the outstanding mechanical properties of the TUPN10 binder were able to effectively alleviate the internal stress caused by the severe volume change of the Si active materials, while maintaining the structural integrity of the Si anode. Obviously, the Si anode with the PVDF binder exhibited a damaged structure with a thickness of 65.75 µm, corresponding to a 332.3% volume expansion. According to previous results, significant cracks in the electrode not only disturb electrical contact, but also continue to generate the SEI layer with the consumption of electrolytes.^[^ ^62^ ^]^

As such, we conducted a comprehensive investigation of the SEI layer formed on the surface of the Si anode with various binders after 100 cycles using an ex situ atomic force microscopy (AFM). As presented in Figure S24 (Supporting Information), the Si anode with the TUPN10 binder exhibited a clear morphology of Si nanoparticles, whereas the Si nanoparticles in the electrode with the PVDF binder were significantly covered by the thick SEI layer owing to the excessive decomposition of electrolytes.^[^ ^63^, ^64^ ^]^ In addition, we verified the broader topography of the Si anodes before and after 100 cycles as 3D mapping images with surface roughness (Figure 4d‒f).^[^ ^65^, ^66^ ^]^ Before cycling, the surface roughness values for Si anodes with TUPN10, TUPN5, and PVDF binders were 63.59, 65.43, and 69.37 nm, respectively. After 100 cycles, however, the surface roughness values were 77.45, 59.38, and 28.11 nm, respectively. For the Si‐TUPN10, clear morphology of silicon nanoparticles was observed even after cycling, but for the Si‐PVDF, it was challenging to identify the initial morphology of the silicon nanoparticles (Figure S24, Supporting Information). We assumed that the increased surface roughness of Si‐TUPN10 could be attributed to slight expansion of the silicon particles during the charging and discharging process, resulting in a somewhat rough surface. Conversely, the significant reduction in surface roughness of Si‐PVDF could be associated with pulverization of the silicon particles following cycling, filling the space through continuous electrolyte decomposition on the electrode surface. These results are also consistent with changes in electrode thickness before and after the cycling (Figure S23, Supporting Information; Figure 4a–c).^[^ ^67^, ^68^, ^69^ ^]^ This is because the thickness of the Si‐PVDF becomes thick due to the pulverization of the silicon particles and the formation of excessively thick SEI layers.

The composition of the SEI layer was further characterized using X‐ray photoelectron spectroscopy (XPS). The high‐resolution C 1s, O 1s, and F 1s spectra of the cycled Si anode with various binders are displayed in Figure 4g‒i, respectively. The C 1s spectra were deconvoluted into four peaks: C─C (284.7 eV), C─O (286.6 eV), C═O (288.4 eV), and Li_2_CO_3_ (289.8 eV).^[^ ^70^ ^]^ The O 1s spectra were fitted by three peaks: C─O (533.7 eV), C═O (532.3 eV), and Li_2_CO_3_ (531.4 eV). Li_2_CO_3_ and O‐containing peaks, which correspond to the decomposition of the carbonate‐based organic electrolyte, were identified in all three Si anodes.^[^ ^70^, ^71^ ^]^ For the F 1s spectra, they were deconvoluted into two peaks: Li_x_PF_y_O_z_ (686.7 eV) and LiF (684.6 eV). When the SEI layers in the Si anode with binders mainly consisted of LiF species, an increase in LiF could lead to the development of stable and effective passivation layers to exhibit a low barrier for Li‐ion diffusion while improving cycling behavior.^[^ ^72^, ^73^ ^]^ In addition, the formation of Li_x_PF_y_O_z_ is derived from the decomposition of carbonate‐based electrolytes and lithium salt, as well as electrochemical reduction.^[^ ^72^ ^]^ Indeed, the change in the SEI component can be largely affected by the physical properties of binders.^[^ ^74^ ^]^ When the binders show weak hydrogen bonds with the Si nanoparticles or have low mechanical properties, they can promote the formation of a thick and unstable SEI layer on the Si anode. As the Si surface is newly exposed from these SEI layers, the electrolyte can be decomposed, which leads to the formation of Li_x_PF_y_O_z_.^[^ ^73^, ^74^ ^]^ It seemed that the formation of Li_x_PF_y_O_z_ in the Si anode with the TUPN10 binder was significantly mitigated compared to the SEI layers of the Si anode with the PVDF binder. Furthermore, the SEI layers in the Si anode with TUPN binders predominantly consisted of LiF species. Due to its enhanced mechanical properties, hydrogen bonding with Si nanoparticles, and Si‐epoxy cross‐linking, the TUPN binder can effectively suppress the pulverization of Si nanoparticles, thereby inducing the formation of a thin and stable SEI layer and mitigating the electrolyte decomposition.^[^ ^74^ ^]^ Overall, the Si anode with TUPN10 binder showed the formation of a thin and high LiF‐containing stable SEI layer by maintaining the integrity of the electrode structure during cycling, which enabled excellent electrochemical performance with enhanced ionic conductivity.

## 3 Conclusion

In summary, a TUPN binder was synthesized via in situ cross‐linking between the TUEG amine and TGIC epoxy groups during the electrode fabrication, for a high‐performance Si anode. Appropriately cross‐linked TUPN10 exhibited outstanding mechanical properties, including tensile strength, extensibility, and resilience, which enables the control of the internal stress generated by volume change during lithiation/delithiation. In addition, the epoxy group of TGIC and the hydroxyl group of Si formed a covalent bond, exhibiting a stable electrolyte–electrode interface with a thin SEI layer. In addition, owing to the thiourea and isocyanurate moieties, the interfacial stability was improved by forming a physical interaction between the current collector and TUPN10 binder. Finally, because of the adequately enriched ratio of oligoether, thiourea, and isocyanurate, the electrolyte wettability of the polar TUPN10 was enhanced, inducing improved electrical conductivity. Based on these versatile characteristics, the Si anode with TUPN10 binder achieved excellent rate performances of 2726.2 mA h g^‒1^ at a current density of 0.2 C and 1839.6 mA h g^‒1^ at a current density of 1.0. The Si anode with the TUPN10 binder exhibited stable cycling stability with a high reversible capacity of 1530.1 mA h g^‒1^ after 100 cycles, which corresponds to a high retention of 73.3%. The design principle of this multifunctional binder provides significant promise for potential applications, to further improve various types of electrode materials.

## 4 Experimental Section

### Materials

1,1′‐Thiocarbonyldiimidazole (TCDI, tech. 90%, Alfa Aesar), 1,2‐bis(2‐aminoethoxy)ethane (EGDA, 98%, Sigma–Aldrich), triglycidyl isocyanurate (TGIC, Sigma–Aldrich), dimethyl suloxide‐d6 (DMSO*‐d*_6_, 99.5 atom % D, Sigma–Aldrich), and *N,N*‐dimethylformamide (DMF, anhydrous, 99.8%, Sigma–Aldrich) were utilized as received. All other reagents and solvents were used as received from standard vendors. Si powder (≈50 nm, Alfa Aesar) was utilized as received. Electrolyte containing 1.0 m LiPF6 in EC/DEC/FEC (= 45/45/10, v/v/v) was purchased from PuriEL.

### Instrumentation and Characterization Techniques

^1^H NMR spectra of TUEGs were recorded on a Bruker Ascend 400 MHz using DMSO‐*d*_6_ as the solvent. Solid‐state ^29^Si cross polarization magic‐angle spinning (CPMAS) nuclear magnetic resonance (NMR) spectra were obtained using 4 mm CPMAS probes and a 500 MHz Bruker ADVANCE III HD NMR spectrometer. Spinning speed and pulse repetition delay of 5 kHz and 5 s were used, respectively. The FT‐IR spectra were recorded on an Agilent 4100 Exoscan FT‐IR spectrometer using attenuated total reflectance (ATR) equipment. Gel fraction and swelling experiments were performed by placing a small piece (ca. 50 mg) of TUPN film into a 20 mL vial filled with electrolyte (1 m LiPF_6_ in EC/DEC (1:1 by volume) + FEC (10%)) or DMF solvent. After storing at 25 °C for 24 h, the gel fraction (*f*_g_) was calculated as

(3) $$f_{g}=W_{a}/W_{d}$$

where *W*_d_ and *W*_a_ denote the weights of the dried film before and after the electrolyte or DMF solvent extraction, respectively. The swelling ratio was calculated as

(4) $$\mathrm{Swelling}\;\mathrm{ratio}=\left(\left(W-W_{0}\right)/W_{0}\right)\times 100\;\left(\%\right)$$

where *W*_0_ and *W* represent the weights before and after electrolyte input, respectively. The thermal stability of the TUPNs was investigated by TGA using a TA Instruments TGA Q5000 under a nitrogen atmosphere. The samples were first heated to 100 °C and maintained for 10 min to evaporate residual water, and then heated to 700 °C at a heating rate of 10 °C min^−1^. DSC was performed using a TA Instruments DSC Q1000 under a nitrogen atmosphere. Samples with a typical mass of 5–10 mg were encapsulated in sealed aluminum pans. They were first equilibrated to −40 °C, heated to 150 °C, and then cooled to −50 °C, which was followed by second heating at a constant rate of 10 °C min^−1^. DMA was performed on a TA Instruments DMA Q800 with an attached cryo accessory using rectangular‐shaped TUPN films (ca. 50 mm (L) × 5 mm (W) × 0.5 mm (T)). DMA was conducted in the film tension mode with a 1‐Hz frequency, 0.01% strain, and 0.001‐N axial force. The specimens were first cooled from 30 to −50 °C and then heated to 100 °C at a constant rate of 5 °C min^−1^ in a nitrogen atmosphere. The cross‐linking density (*ν*_e_) of the TUPNs was calculated as

(5) $$\nu_{e}=E^{\prime }/3\mathrm{RT}$$

where *E’*, *R*, and *T* denote the storage modulus, universal gas constant, and absolute temperature in the rubbery region (*ca*. 356.15 K), respectively. Uniaxial tensile testing was conducted on an Instron LR5K universal testing machine (UTM, Lloyd Instruments) at a strain rate of 0.008 s^−1^ (10 mm min^−1^), unless otherwise noted. Rectangular‐shaped tensile bars (*ca*. 40 mm (L) × 5 mm (W) × 0.5 mm (T), gauge length = 20 mm) were stamped out from the films using a cutting die. Cyclic tensile testing was performed on the same UTM using rectangular‐shaped TUPN films at a strain rate of 0.008 s^−1^ (10 mm min^−1^). All tensile tests were performed at room temperature (23 °C) and specified humidity (25 ± 1 °C, RH 45 ± 8% in air), unless otherwise noted. The ionic conductivity was measured by the EIS method and calculated as

(6) σ=l/RA

where *σ*, *R*, *l*, and *A* denote the ionic conductivity, bulk resistance obtained from the EIS plot, thickness of the film, and measured surface area, respectively. AFM was conducted using an NX10 instrument. The chemical composition of the anode surface was investigated by XPS using an Axis Supra instrument.

### Synthesis of Poly(ether‐thiourea) with Triethylene Glycol as a Spacer (TUEG)

Poly(ether‐thiourea)s with triethylene glycol as a spacer were designated as TUEG#, where # denotes the molecular weight estimated by ^1^H NMR end group analysis. TUEGs were synthesized similarly to the route described in previous literature (Figure S1, Supporting Information).^[^ ^27^ ^]^ The preparation procedure was conducted under a nitrogen atmosphere in a glove box (<0.1 ppm of H_2_O and O_2_). For the synthesis of TUEG2600, EGDA (4.4 g, 30 mmol) and DMF (14 mL) were placed into a 100 mL round‐bottomed flask with a magnetic stirring bar, and TCDI (5.0 g, 28 mmol) was added into the flask under stirring. The reaction flask, sealed with a stopper, was transferred to a thermostatic oil bath at 25 °C outside the glove box. After stirring for 24 h, the solution was diluted with chloroform (15 mL) and precipitated into an excess of ether (800 mL). The dissolution‐precipitation procedure was repeated thrice. The resulting precipitate was subsequently dried under vacuum conditions at 120 °C overnight, yielding a light brownish solid (4.8 g, 85%). TUEG4300 and TUEG1000 were also prepared via the same procedure, but different ratios of the TCDI and EGDA monomer charge ([TCDI]: [EGDA]) of 1:1.06 and 1:1.40, respectively, were adopted. ^1^H NMR of TUEGs (400 MHz, DMSO‐*d*_6_, δ/ppm, tetramethylsilane (TMS) ref) (Figure S2, Supporting Information):7.54 (br, C(S)N*H*), 3.60‐3.20 (br, C*H*_2_O, C(S)NHC*H*_2_), 2.68 (t, C*H*_2_NH_2_). The degree of polymerization (DP) was determined from the intensity ratio between the peaks at 7.54 (br, C(S)NH) and 2.68 (t, C*H*_2_NH_2_) ppm. The molecular weight was obtained by multiplying DP by a repeating unit (190.26 g mol^−1^) and then adding the molecular weight of the terminal group (148.21 g mol^−1^).

### Preparation of Thiourea Polymer Network (TUPN) Films

Cross‐linked TUEG‐TGIC films were designated as TUPN*#*, where *#* denotes the weight percentage (wt.%) of TGIC in the TUPN. The following procedure was adopted for the preparation of the TUPN10. TUEG2600 (0.70 g, 0.27 mmol) and TGIC (0.08 g, 0.27 mmol) in DMF (3.5 mL) were cast onto a Teflon plate (7 × 3 cm^2^), followed by drying on a hot plate at 50 °C overnight. After subsequent drying at 80 °C under vacuum conditions for 1 h, the resultant film on a Teflon plate was cured at 120 °C for 2 h and post‐cured at 140 °C for 2 h under vacuum the same conditions. TUPN5 and TUPN23 were prepared using the same procedure, except that TUEG4300 and TUEG1000 were used for TUPN5 and TUPN23, respectively. The molar feed ratio of the amine end group of TUEG to the epoxy group of TGIC ([amine]: [epoxy]) was fixed at 1:1.5 for all the films; this was for the complete reaction of the primary amine and epoxy groups without any unreacted, residual primary amine end‐groups or epoxy functional groups. The thickness of the TUPNs was approximately 0.5 mm. All the films were additionally dried at 50 °C under vacuum conditions for 24 h before analyses.

### Preparation of Si‐TUPN Anode

The Si‐TUPN anode was prepared by stirring a homogeneous slurry of 60 wt.% Si powder, 20 wt.% TUPN binder, and 20 wt.% Super P in N‐methyl‐2‐pyrrolidone (NMP) solvent. The slurry was coated onto a copper current collector via doctor‐blading, and then dried for three steps (120 and 140 °C for 2 h, respectively, and 80 °C overnight) in a vacuum oven, simultaneously with the in situ polymerization of the TUPN binder. For comparison, a Si‐PVDF anode was prepared with a PVDF binder using the same procedure.

### Electrochemical Characterization

The as‐prepared anode was assembled into a 2032‐type coin cell with a Li foil and a Celgard 2400 separator in an Ar‐filled glove box. The electrolyte contained 1 m LiPF6 in EC/DEC (1:1 by volume) with 10 wt.% FEC as an additive. For the full‐cell test, the Si anode was pre‐cycled once at a current density of 0.1 A g^−1^, and the pre‐cycled Si electrode with a mass loading of 0.6 mg cm^−2^ and a reversible capacity of 1.56 mAh cm^−2^ was coupled with NCM_622_ cathode (mass loading of 8.35 mg cm^−2^, reversible specific capacity of 170 mAh g^−1^ at 1.0 C, and reversible areal capacity of 1.42 mAh cm^−2^) with an N/P ratio of 1.10. The full‐cell was subjected to cycling within the voltage range of 2.7 to 4.3 V, at a current density of 0.5 C at room temperature. The electrochemical performance of the cells was measured using an automatic battery cycler (WBCS3000, WonATech Co.) in a cut‐off voltage of 0.01‒1.2 V. All test cells were activated at 0.1 A/g in the initial two cycles. CV was performed with a BCS‐805 (Bio‐Logic Co.) instrument. EIS was conducted using an SP‐150 (Bio‐Logic Co.) workstation in the frequency range of 1.00–0.1 Hz. The post‐cycled electrodes were analyzed by disassembling them in an Ar‐filled glove box and washing them with dimethyl carbonate (DMC).

## Conflict of Interest

The authors declare no conflict of interest.

## Supporting information

Supporting Information

## Data Availability

Research data are not shared.
