# Supplementary material for: A Multifunctional Interlocked Binder with Synergistic In Situ Covalent and Hydrogen Bonding for High‐Performance Si Anode in Li‐ion Batteries
Source: Adv Sci (Weinh). 2023 Aug 16;10(30):2302144. doi: 10.1002/advs.202302144 (PMC10602578; doi:10.1002/advs.202302144)
Supplement: Supplementary file 1 — Supporting Information [file ADVS-10-2302144-s001.pdf]

ADVANCED SCIENCE
Open Access

Supporting Information

for *Adv. Sci.*, DOI 10.1002/advs.202302144

A Multifunctional Interlocked Binder with Synergistic In Situ Covalent and Hydrogen Bonding for High-Performance Si Anode in Li-ion Batteries

Jae Hyuk Hwang, Eunji Kim, Eun Young Lim, Woohwa Lee, Ji-Oh Kim, Inhye Choi, Yong Seok Kim, Dong-Gyun Kim, Jin Hong Lee* and Jong-Chan Lee**

Supporting Information for

A Multifunctional Interlocked Binder with Synergistic In-Situ Covalent and Hydrogen Bonding for High-Performance Si Anode in Li-ion Batteries

Jae Hyuk Hwang,[‡ab] *Eunji Kim,*[‡c] *Eun Young Lim,*[c] *Woohwa Lee,*[a] *Ji-Oh Kim,*[c] *Inhye Choi,*[c] *Yong Seok Kim,*[ad] *Dong-Gyun Kim,*[*ad] *Jin Hong Lee,*[*c] *and Jong-Chan Lee*[*b]

[a] Advanced Materials Division, Korea Research Institute of Chemical Technology, 141 Gajeong-ro, Yuseong-gu, Daejeon 34114, Republic of Korea.

[b] School of Chemical and Biological Engineering and Institute of Chemical Processes, Seoul National University, 599 Gwanak-ro, Gwanak-gu, Seoul 08826, Republic of Korea.

[c] School of Chemical Engineering, Pusan National University, 2, Busandaehak-ro 63beon-gil, Geumjeong-gu, Busan 46421, Republic of Korea.

[d] Advanced Materials and Chemical Engineering, KRICT School, University of Science and Technology, 217 Gajeong-ro, Yuseong-gu, Daejeon 34114, Republic of Korea.

‡J. H. Hwang and E. Kim contributed equally to this work.

*Correspondence to jinhong.lee@pusan.ac.kr (Prof. Jin Hong Lee), dgkim@krict.re.kr (Dr. Dong-Gyun Kim), and jongchan@snu.ac.kr (Prof. Jong-Chan Lee)

Table of Contents

1. Synthesis of TUEG S2
2. Synthesis of Thiourea Polymer Network (TUPN) S4
3. Rheological, Thermal, and Mechanical Characterization of TUPN S6
4. Electrochemical Characterization of TUPN binder S12

Synthesis of TUEG

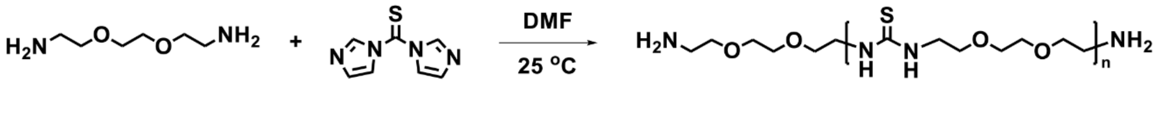

Figure S1. Synthesis of TUEG.

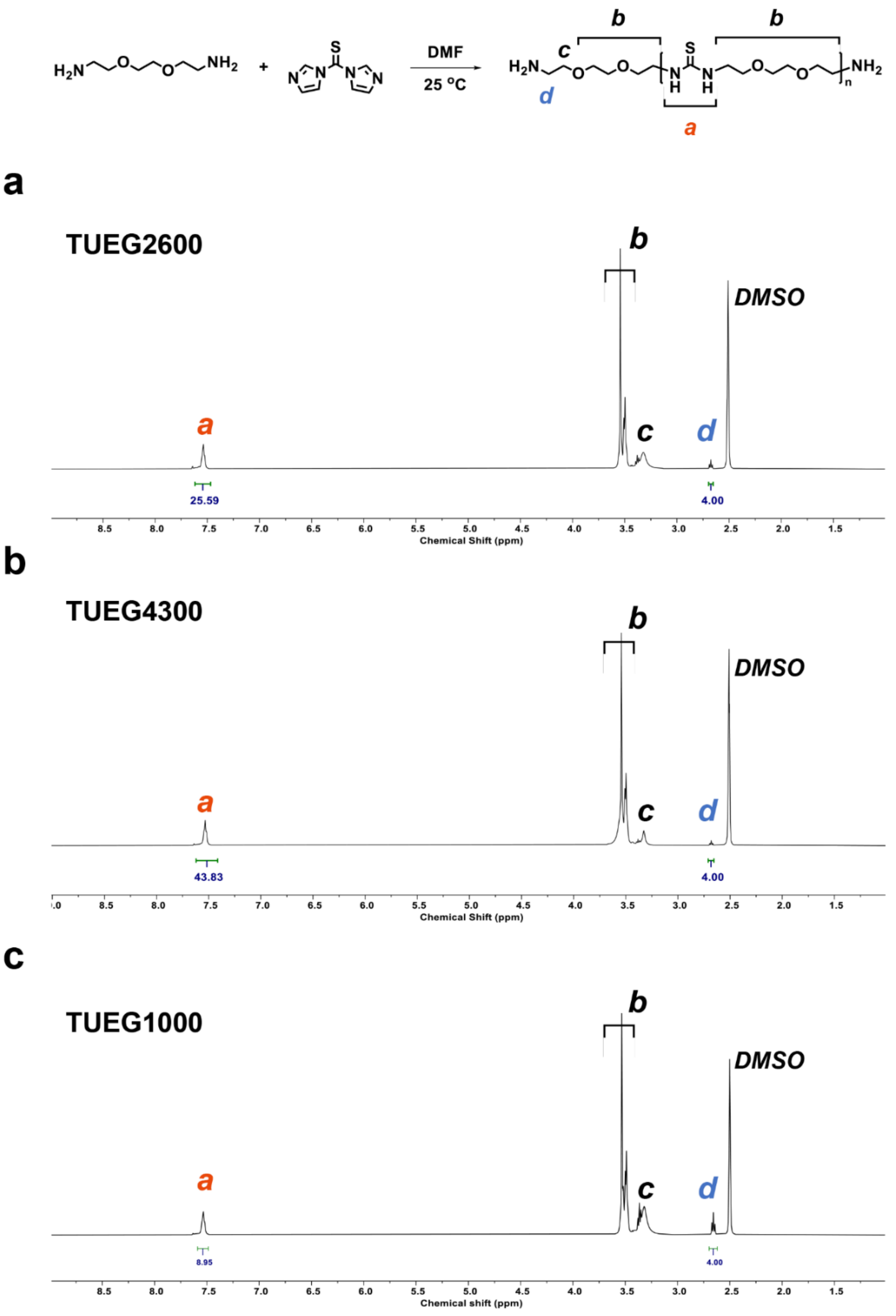

Figure S2. ^{1}H NMR spectra of (a) TUEG2600, (b) TUEG4300, and (c) TUEG1000.

Synthesis of Thiourea Polymer Network (TUPN)

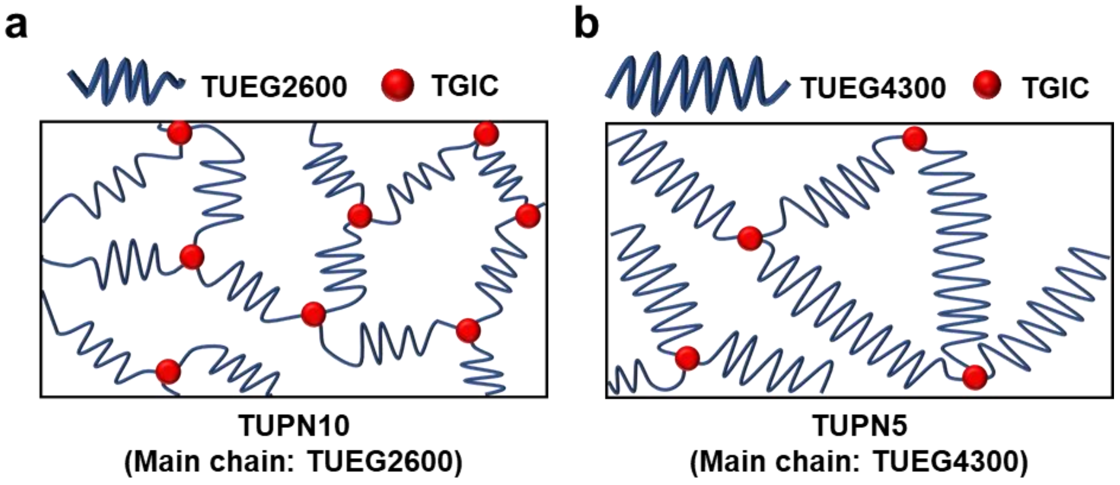

Figure S3. Schematic illustrations of the different cross-linking densities of (a) TUPN10 and (b) TUPN5 according to the chain length of TUEG2600 and TUEG4300, respectively.

Table S1. Composition, gel fraction, cross-linking density of TUPN10 and TUPN5.

Sample	TUEG sample	W_{TGIC}[a]	v_e[b] (mol m^{-3})	$f_{g,ETL}$[c]	$f_{g,DMF}$[d]
TUPN 10	TUEG2600	0.10	123	0.97	0.88
TUPN 5	TUEG4300	0.05	103	0.89	0.79

[a] Weight fraction of TGIC in the TUPNs. [b] Cross-linking density, calculated by $v_e = E'/3RT$, where E', R, and T are the storage modulus, universal gas constant, and absolute temperature in the rubbery region (ca. 356.15 K), respectively. [c] Gel fraction, obtained by $f_{g,ETL} = W_{a,ETL}/W_{d,ETL}$, where $W_{d,ETL}$ and $W_{a,ETL}$ are the weights of dried film before and after the electrolyte (1 M LiPF6 in EC/DEC (1:1 by volume) with 10 wt % FEC) extraction. [d] Gel fraction, obtained by $f_{g,DMF} = W_{a,DMF}/W_{d,DMF}$, where $W_{d,DMF}$ and $W_{a,DMF}$ are the weights of dried film before and after *N,N*-dimethylformamide (DMF) solvent extraction.

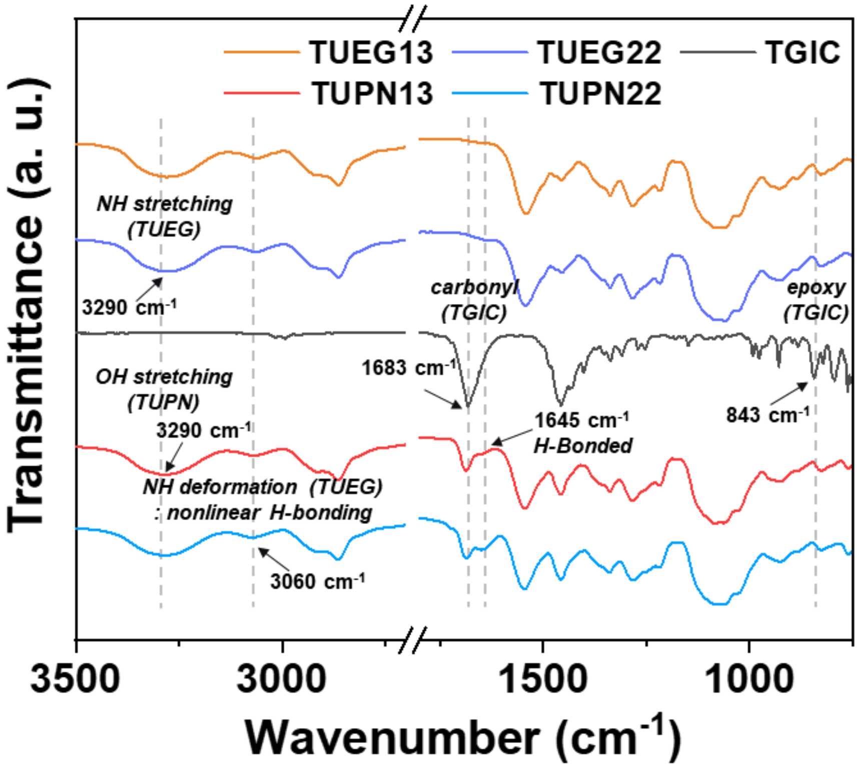

Figure S4. FT-IR spectra of TUEG2600, TUEG4300, TGIC, TUPN10, and TUPN5.

Rheological, Thermal, and Mechanical Characterization of TUPN

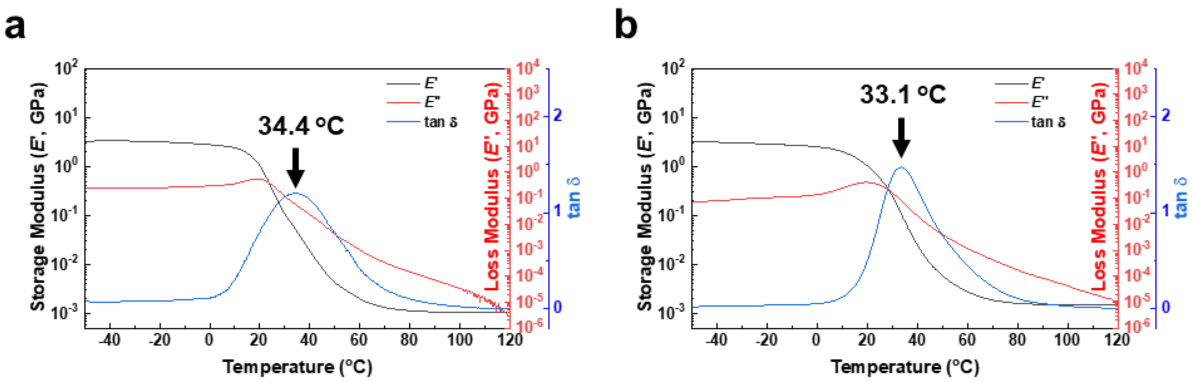

Figure S5. DMA curves of (a) TUPN10 and (b) TUPN5 at a constant frequency of 1 Hz and a heating rate of 5 °C min^{-1}.

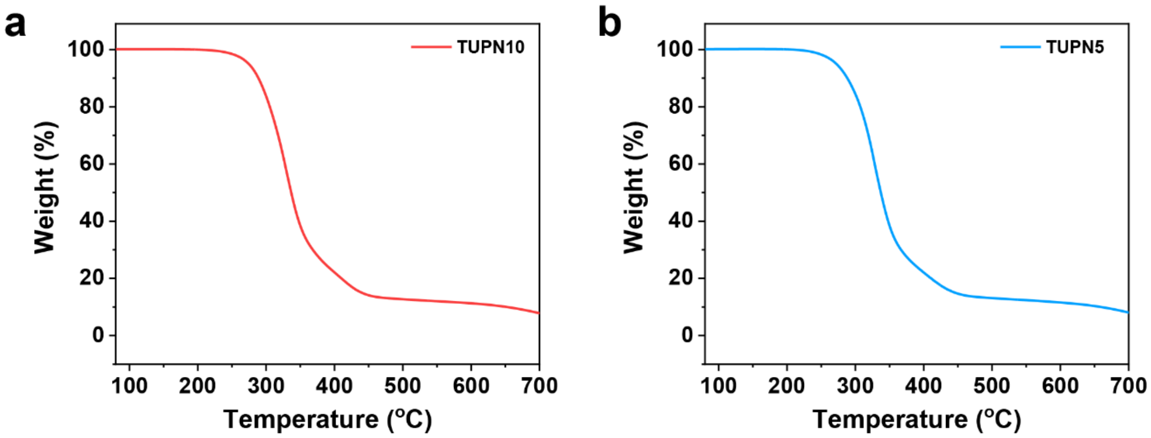

Figure S6. TGA curves of (a) TUPN10 and (b) TUPN5.

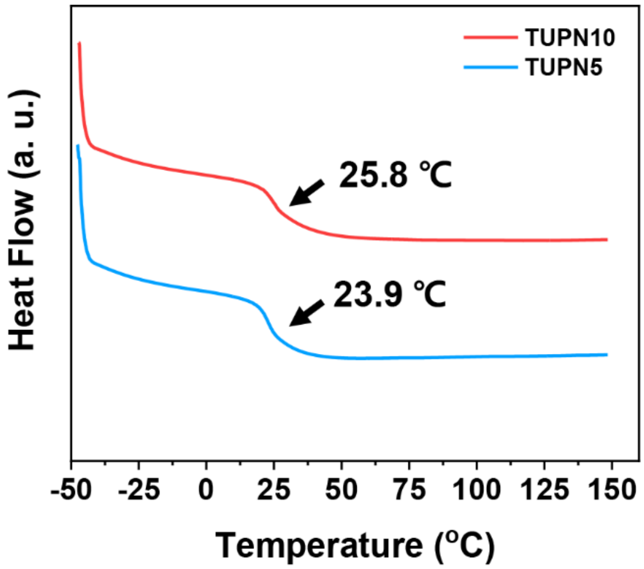

Figure S7. DSC thermograms of TUPN10 and TUPN5.

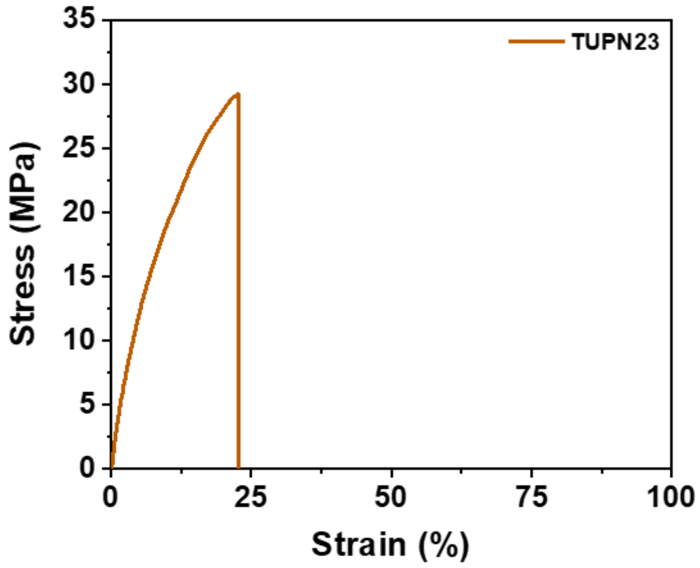

Figure S8. Representative stress−strain curves of TUPN23 at room temperature (25±1 °C) and a strain rate of 0.008 s^{-1}.

Table S2. Summary of mechanical properties of TUPN10 and TUPN5.

Sample	E^a (MPa)	$\sigma_y{}^b$ (MPa)	$\varepsilon_b{}^c$ (%)	$U_T{}^d$ (MJ m^{-3})
TUPN 10	183 ± 30	11.5 ± 2.4	286 ± 32	15.7 ± 2.9
TUPN 5	68 ± 10	4.1 ± 0.6	338 ± 26	9.2 ± 1.6

Determined from tensile testing at room temperature (25±1 °C) and a strain rate of 0.008 s^{-1}. E, σ_y, ε_b, and U_T are [a] elastic modulus, [b] yield stress, [c] strain at break, and [d] toughness, respectively.

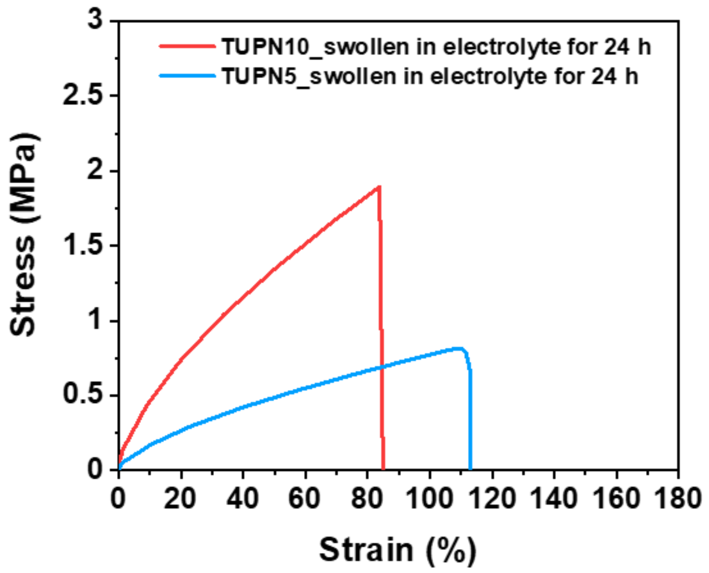

Figure S9. Uniaxial stress−strain curves of TUPN10 and TUPN5 at room temperature (25±1 °C) and a strain rate of 0.008 s^{-1} after electrolyte swelling for 24 h.

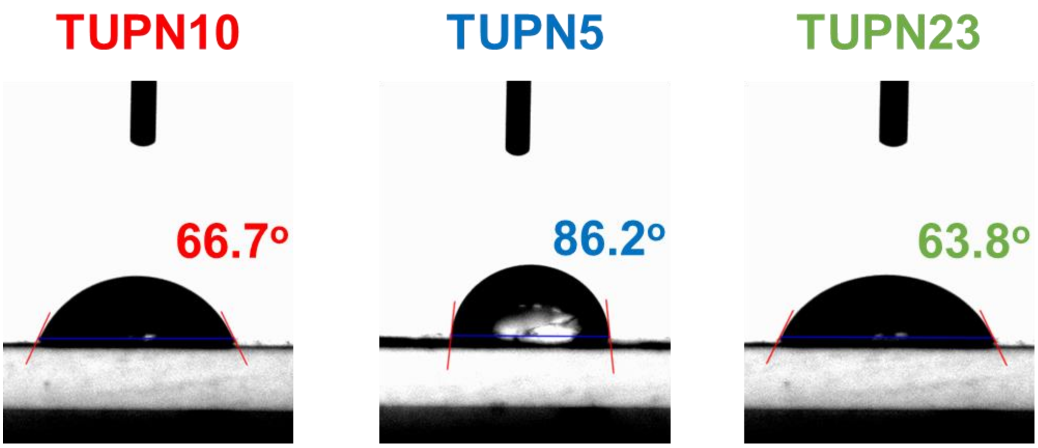

Figure S10. The water contact angles of TUPN films.

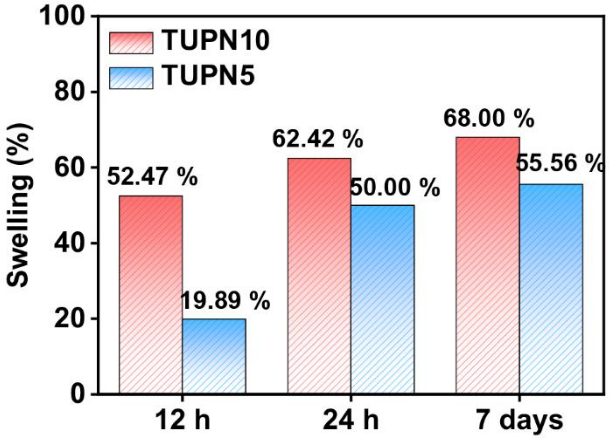

Figure S11. The swelling ratio of TUPN10 and TUPN5 in electrolyte.

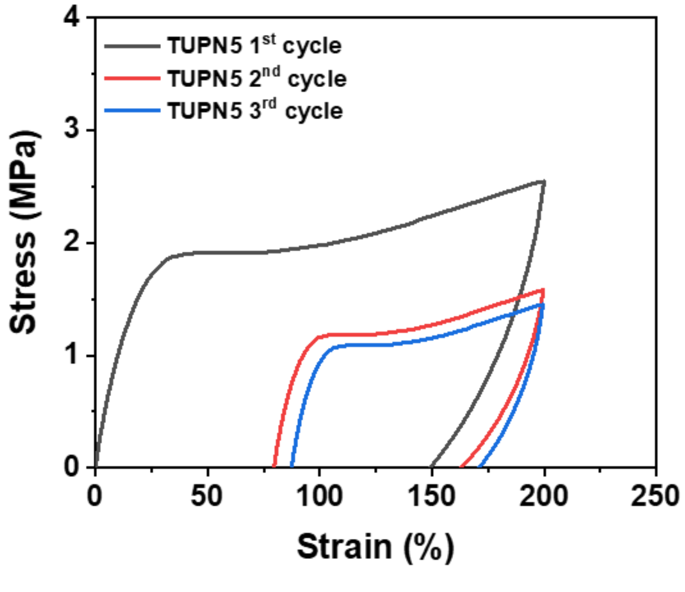

Figure S12. Cyclic stress-strain curves of TUPN5 (~ 200% strain) at room temperature (25±1 ℃) and a strain rate of 0.008 s^{-1}.

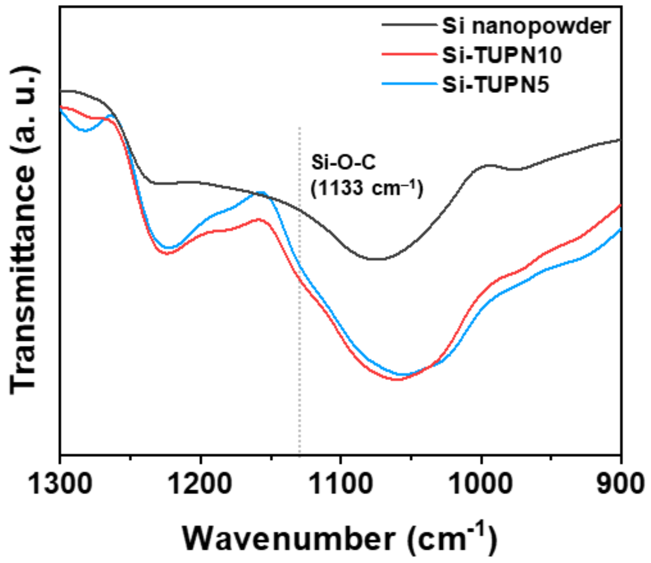

Figure S13. FT-IR spectra of Si nanopowder, Si-TUPN10, and Si-TUPN5.

Table S3. Gel fraction of Si-TUPN10 and Si-TUPN5.

Sample	$f_{g,ETL}$[a]	$f_{g,DMF}$[b]
Si-TUPN10	0.99	0.96
Si-TUPN5	0.97	0.94

[a] Gel fraction, obtained by $f_{g,ETL} = W_{a,ETL}/W_{d,ETL}$, where $W_{d,ETL}$ and $W_{a,ETL}$ are the weights of dried chunk composite before and after the electrolyte (1 M $LiPF_6$ in EC/DEC (1:1 by volume) with 10 wt % FEC) extraction. [b] Gel fraction, obtained by $f_{g,DMF} = W_{a,DMF}/W_{d,DMF}$, where $W_{d,DMF}$ and $W_{a,DMF}$ are the weights of dried chunk composite before and after *N,N*-dimethylformamide (DMF) solvent extraction.

Electrochemical Characterization of TUPN binder

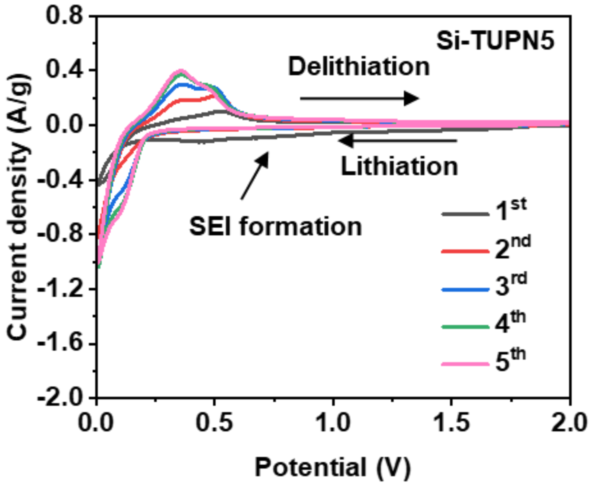

Figure S14. CV curve of the Si-TUPN5 electrode at scan rate of 0.1 mV s^{-1}.

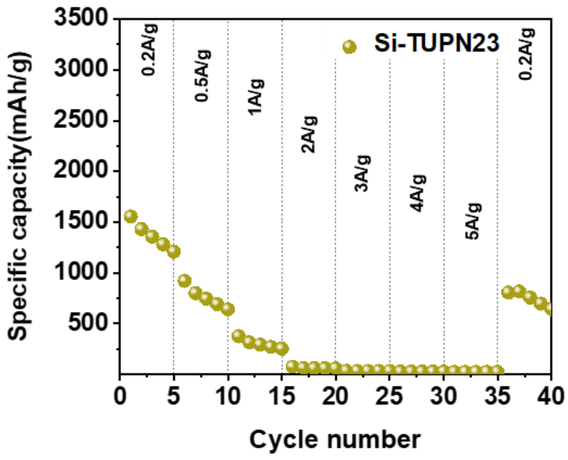

Figure S15. Rate performance of Si-TUPN23 electrode with various C-rate.

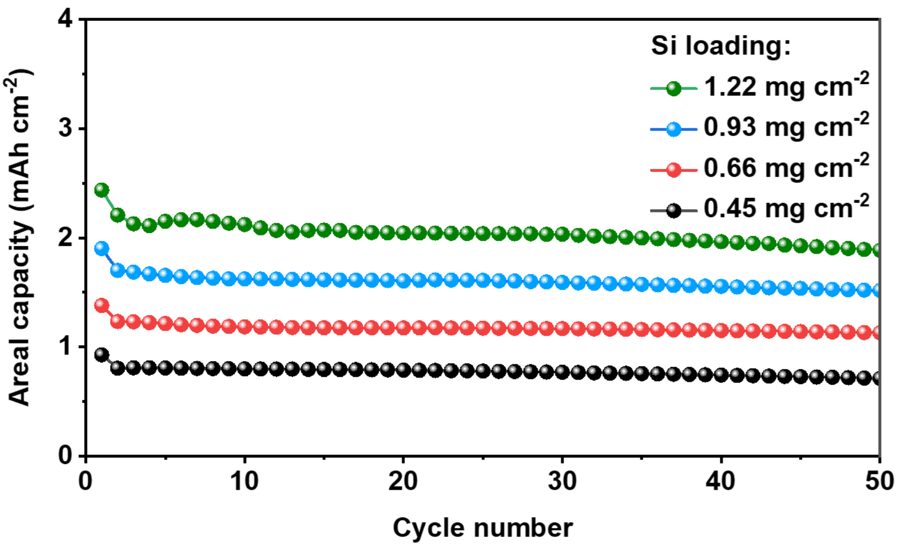

Figure S16. Areal capacity of Si anode with TUPN10 evaluated at different active material mass loading under a current density of 1.0 A g^{-1}.

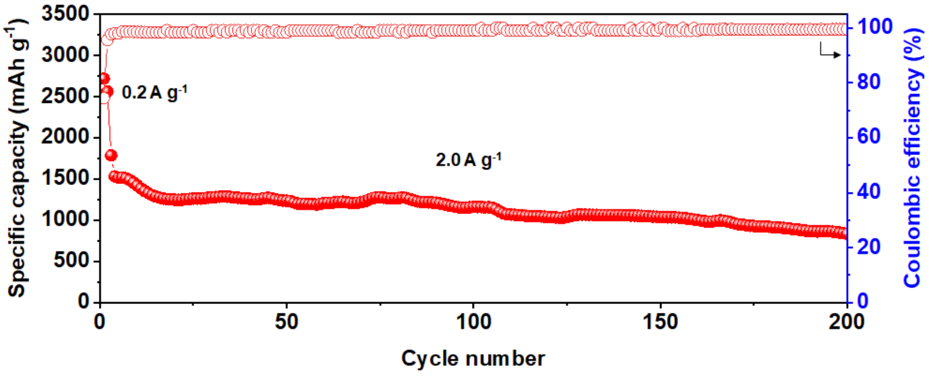

Figure S17. Cycling performance at 2.0 A g^{-1} of the Si anode with TUPN10 binder.

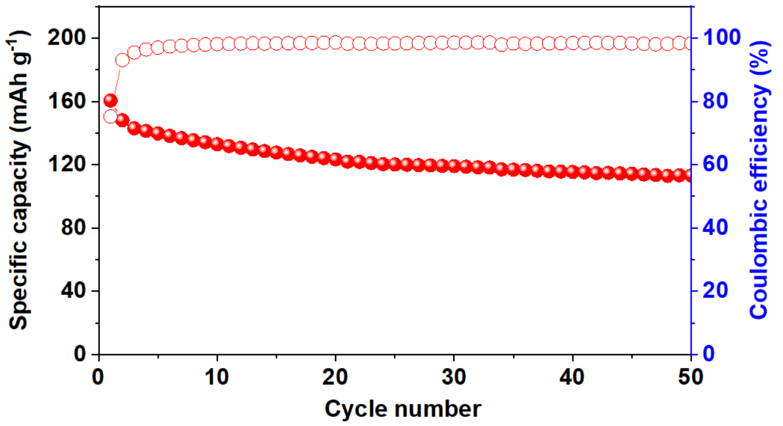

Figure S18. Cyclic performance of a full-cell using NCM_{622} electrode as the cathode and Si with TUPN10 binder as the anode.

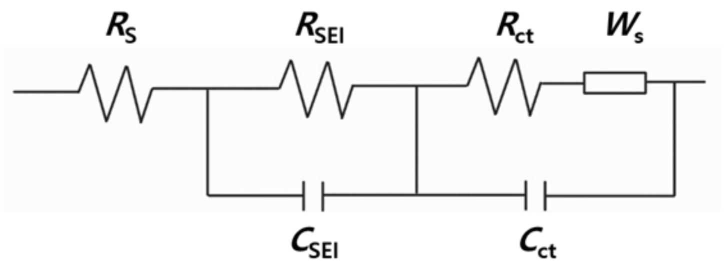

Figure S19. Equivalent circuit model of cycled cells.

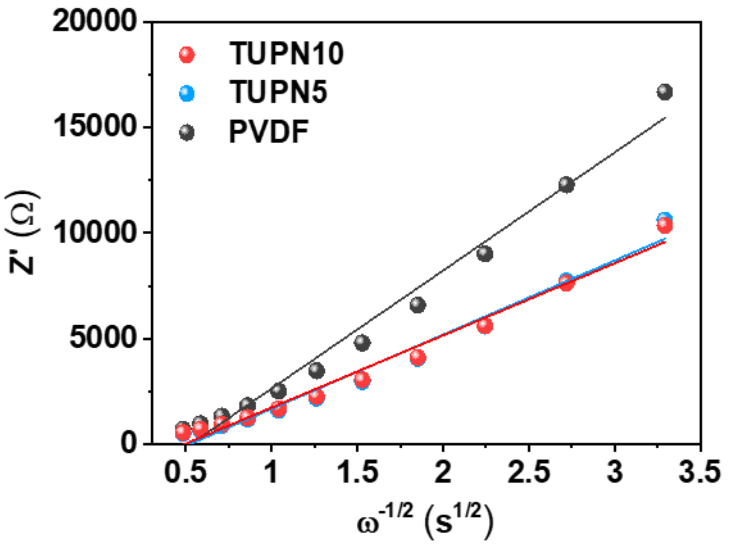

Figure S20. Linear relationship between *Z'* and $\omega^{-1/2}$ in the low-frequency region.

Table S4. Calculated Li-ion diffusion (D_{Li^+}) coefficient of the Si anode with various binders.

Sample	D_{Li^+}
TUPN 10	2.38×10^{-21}
TUPN 5	2.25×10^{-21}
PVDF	8.89×10^{-22}

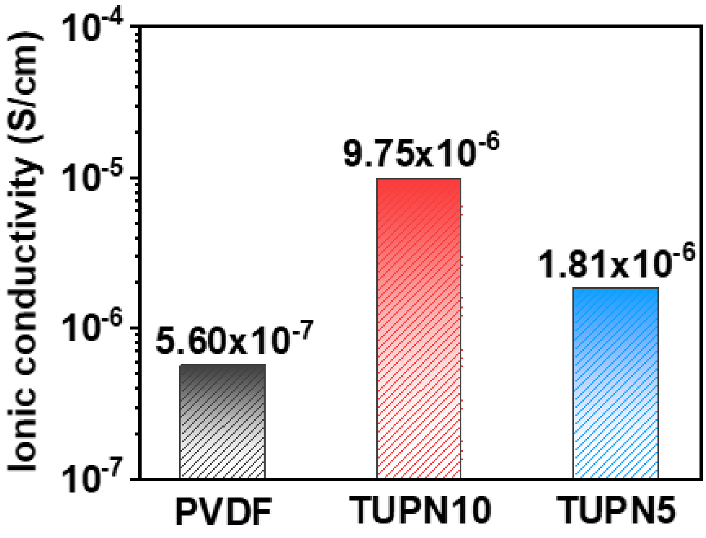

Figure S21. Ionic conductivity values of PVDF, TUPN10, and TUPN5 films, respectively.

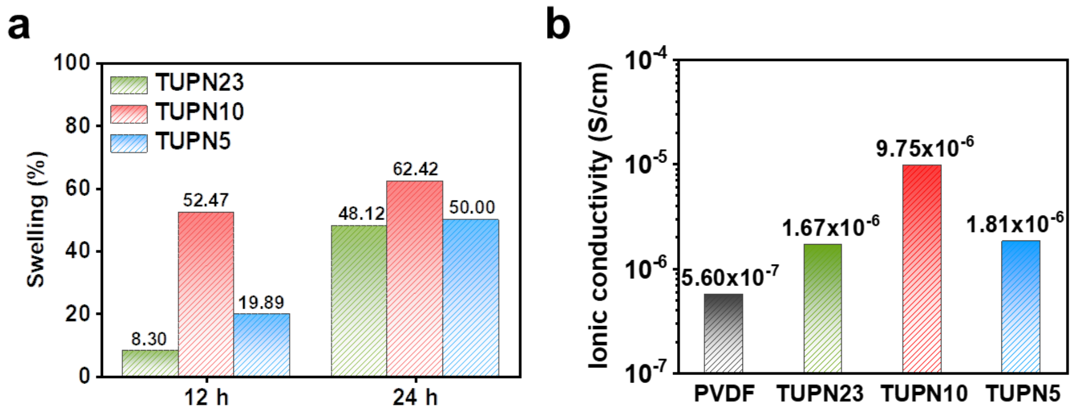

Figure S22. Comparison of (a) swelling ratio in electrolyte and (b) ionic conductivity values of TUPN10, TUPN5, and TUPN23.

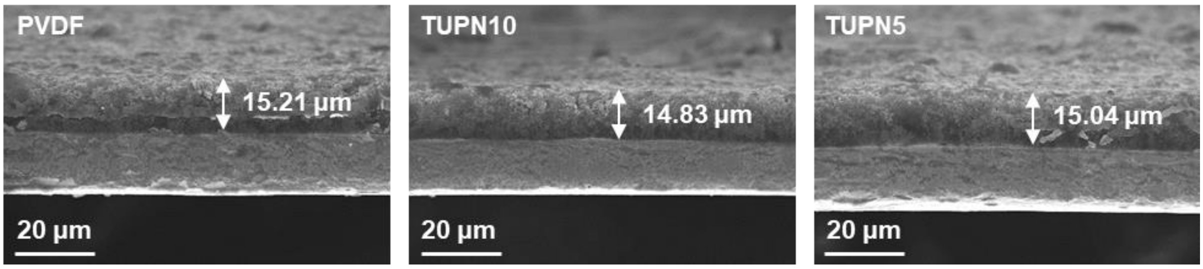

Figure S23. Cross-section and top surface SEM image of the Si anodes with TUPN10, TUPN5, and PVDF binders before cycling.

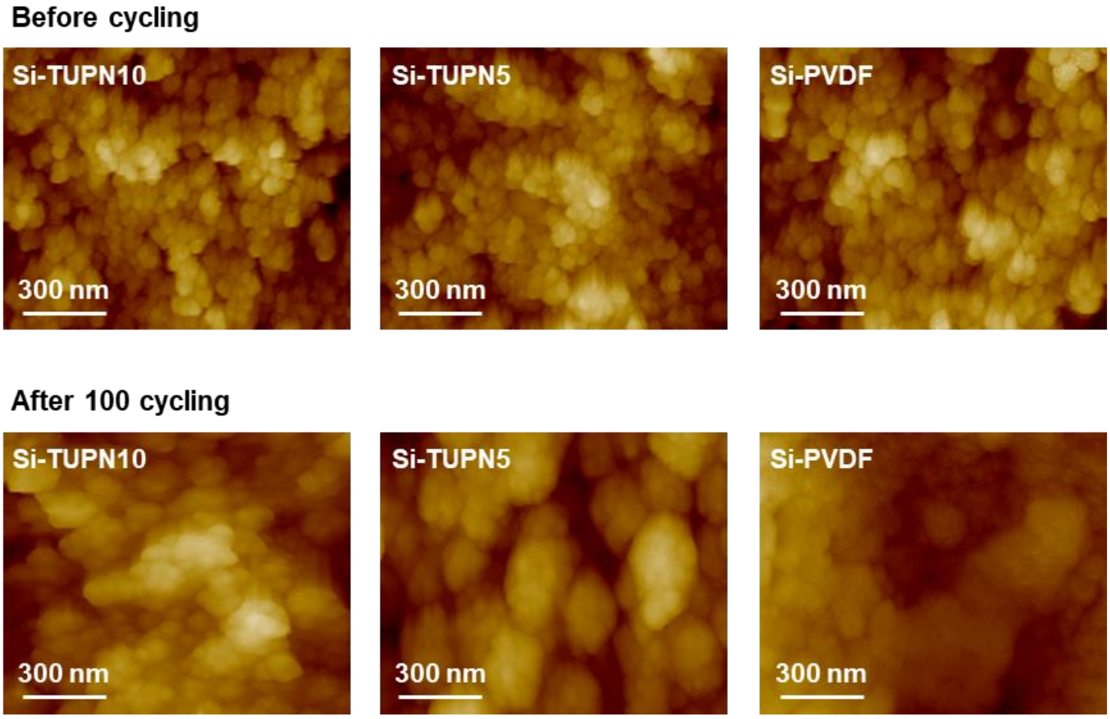

Figure S24. Top surface AFM images of the Si anodes with TUPN10, TUPN5, and PVDF binders before and after cycling.
